# Clinical performance among recent graduates in nine low‐ and middle‐income countries

**DOI:** 10.1111/tmi.13224

**Published:** 2019-03-24

**Authors:** Todd P. Lewis, Sanam Roder‐DeWan, Address Malata, Youssoupha Ndiaye, Margaret E. Kruk

**Affiliations:** ^1^ Department of Global Health and Population Harvard T.H. Chan School of Public Health Boston MA USA; ^2^ Ifakara Health Institute Dar es Salaam Tanzania; ^3^ Malawi University of Science and Technology Limbe Malawi; ^4^ Planning, Research, and Statistics Ministry of Health and Social Action Dakar Senegal

**Keywords:** quality of care, clinical performance, low‐ and middle‐income countries, qualité des soins, performance clinique, pays à revenu faible ou intermédiaire

## Abstract

**Objectives:**

Recent studies have identified large and systematic deficits in clinical care in low‐income countries that are likely to limit health gains. This has focused attention on effectiveness of pre‐service education. One approach to assessing this is observation of clinical performance among recent graduates providing care. However, no studies have assessed performance in a standard manner across countries. We analysed clinical performance among recently graduated providers in nine low‐ or middle‐income countries.

**Methods:**

Service Provision Assessments from Haiti, Kenya, Malawi, Namibia, Nepal, Rwanda, Senegal, Tanzania, and Uganda were used. We constructed a Good Medical Practice Index that assesses completion of essential clinical actions using direct observations of care (range 0–1), calculated index scores by country and clinical cadre, and assessed the role of facility and clinical characteristics using regression analysis.

**Results:**

Our sample consisted of 2223 clinicians with at least one observation of care. The Good Medical Practice score for the sample was 0.50 (SD = 0.20). Nurses and midwives had the highest score at 0.57 (SD = 0.20), followed by associate clinicians at 0.43 (SD = 0.18), and physicians at 0.42 (SD = 0.16). The average national performance varied from 0.63 (SD = 0.18) in Uganda to 0.39 (SD = 0.17) in Nepal, persisting after adjustment for facility and clinician characteristics.

**Conclusions:**

These results show substantial gaps in clinical performance among recently graduated clinicians, raising concerns about models of clinical education. Competency‐based education should be considered to improve quality of care in LMICs. Observations of care offer important insight into the quality of clinical education.

## Abstract (fr)

**Objectifs:**

Des études récentes ont identifié des déficits importants et systématiques dans les soins cliniques dans les pays à faible revenu, susceptibles de limiter les progrès en matière de santé. Cela a attiré l'attention sur l'efficacité de la formation initiale. Une des approches permettant cette évaluation a été l'observation de la performance clinique des diplômés récents dispensant des soins. Cependant, aucune étude n'a évalué la performance de manière standard dans tous les pays. Nous avons analysé les performances cliniques de prestataires récemment diplômés dans neuf pays à revenu faible ou intermédiaire.

**Méthodes:**

Des évaluations de la fourniture de services en Haïti, au Kenya, au Malawi, en Namibie, au Népal, au Rwanda, au Sénégal, en Tanzanie et en Ouganda ont été utilisées. Nous avons construit un indice de bonne pratique médicale qui évalue l'achèvement des actions cliniques essentielles à l'aide d'observations directes des soins (plage de 0 à 1), calculé les scores d'index par pays et par cadre clinique, et évalué le rôle de l’établissement et des caractéristiques cliniques à l'aide d'une analyse de régression.

**Résultats:**

Notre échantillon était constitué de 2.223 cliniciens avec au moins une observation des soins. Le score de bonne pratique médicale pour l’échantillon était de 0,50 (écart‐type = 0,20). Les infirmières et les sages‐femmes avaient le score le plus élevé de 0,57 (écart‐type = 0,20), suivies des cliniciens associés avec 0,43 (écart‐type = 0,18) et des médecins avec 0,42 (écart‐type = 0,16). La performance moyenne nationale variait de 0,63 (écart‐type = 0,18) en Ouganda à 0,39 (écart‐type = 0,17) au Népal, persistant après ajustement en fonction des caractéristiques de l’établissement et du clinicien.

**Conclusions:**

Ces résultats montrent des écarts substantiels dans les performances cliniques des cliniciens récemment diplômés, ce qui soulève des inquiétudes concernant les modèles de formation clinique. Une éducation axée sur les compétences devrait être envisagée pour améliorer la qualité des soins dans les PRFM. Les observations des soins offrent des informations importantes sur la qualité de la formation clinique.

## Introduction

In recent years, governments and global health actors have committed to achieving universal health coverage (UHC), aiming to improve health outcomes and increase financial risk protection for all people. While an essential step forward, the UHC movement has placed less emphasis on quality of care. However, poor quality may limit the beneficial impacts of UHC, particularly in low‐ and middle‐income countries (LMICs), where baseline quality standards are often not met. Evidence indicates that overall quality of care is low and varies both across and within countries, even for basic maternal and child health services 1, 2, 3. To reap the benefits of increased coverage, commensurate improvements in quality are required.

One important element of a high‐quality health system is a competent health workforce, and many governments have dedicated resources to strengthening human resources for health. However, these efforts have largely focused on expanding health worker numbers and improving workforce distribution 4, 5. National health workforce efforts rarely emphasise quality of care 6, 7, and little is known about the quality of services new clinicians in LMICs are providing. When governments do seek to improve health worker performance, efforts focus on in‐service training for clinicians already in practice. Studies have found that such training has only a modest impact on quality and cannot by itself close the large quality deficits observed 8, 9.

One contributing factor to poor observed quality of care may be weak health professions education, the course of studies that prepares a health care worker for entry into practice 10. Past studies have noted that while professional demands on health care workers continue to increase, educational systems are failing to keep pace, resulting in ‘the mismatch of professional competencies to patient and population priorities … producing ill‐equipped graduates from under financed institutions 11, 12.’ Health professions education is hampered by outdated curricula and pedagogy, poor adaption to local contexts, insufficient systems for ensuring educational quality, and a dearth of qualified tutors and clinical teachers 11, 13, 14, 15. Schools note a lack of qualified students for training, congestion at clinical placement sites, limited mentorship, inadequate equipment and technology, and difficulties with faculty recruitment and retention. Students face issues of inadequate housing, transportation, and classroom space 15, 16. Furthermore, existing programmes often do not emphasise the importance of quality of care in their curricula 17, 18.

This study assesses the performance of recent clinical graduates in completing fundamental clinical skills in practice in nine low‐ and middle‐income countries. To this end, we constructed a Good Medical Practice Index, a set of essential clinical items required to make a correct diagnosis and provide appropriate treatment in three primary care service areas: (i) antenatal care, (ii) family planning, and (iii) care of sick children and compare performance across provider types and countries. Results can be used to inform efforts to improve clinical education and other strategies to improve quality of care.

## Methods

### Study sample

Data for each country were obtained from Service Provision Assessments (SPA), surveys of health facilities conducted by the Demographic and Health Surveys Program. The SPA includes an audit of facility resources, surveys on clinical practices, and direct observations of antenatal care, family planning and sick child care. SPA surveys occurred at different times across countries; some countries, such as Namibia and Malawi, have only conducted one survey, while other countries have conducted surveys multiple times, such as Tanzania in 2006 and 2015, or survey continuously, as Senegal has since 2012. The most recent available SPA data were used for each country, including Haiti, 2013; Kenya, 2010; Malawi, 2013; Namibia, 2009; Nepal, 2015; Rwanda, 2007; Senegal, an ongoing survey from 2013 to 2015; Tanzania, 2015; and Uganda, 2007. Several other countries have conducted SPA surveys but are not included in this analysis due to age of the data, existence of a more recent survey from the same country, or data inaccessibility. The surveys use nationally representative samples, and censuses or near censuses (in Malawi, Namibia, and Rwanda) of a nation's health facilities. Within surveyed health facilities, up to five patients per provider per clinical area were selected for observation using systematic random sampling. Trained observers assessed first visits or follow‐up visits in their entirety for antenatal care, family planning consultations, and sick child care consultations for children aged five years or younger. To assess the quality of pre‐service education, we analysed the performance of providers in the first three years of practice post graduation. Providers were grouped into four categories: physicians, associate clinicians (e.g. clinical officers), nurses and midwives (e.g. registered nurses, nurse midwives), and other providers (e.g. counsellors, social workers) (Appendix Table A1). Analyses did not include those in the ‘other providers’ category, as the education these providers receive varies greatly both between and within countries.

### Outcome definition and assessment

**Figure 1:**
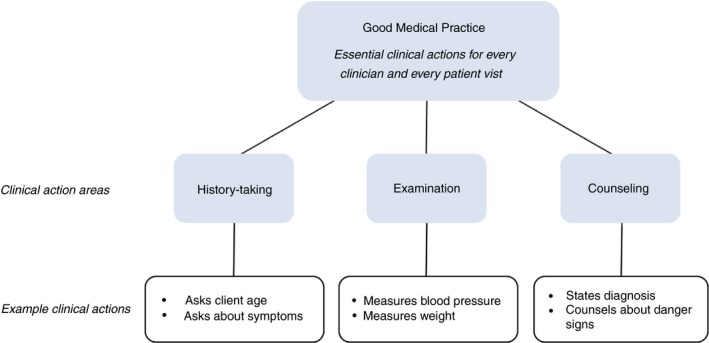
Conceptual model of Good Medical Practice for every patient encounter. [Colour figure can be viewed at http://wileyonlinelibrary.com]

We developed the Good Medical Practice Index (GMPI) to assess the minimum clinical performance in assessing the patient that is essential for making a diagnosis and proposing correct management and that is expected of all clinicians providing clinical care across visit types (Figure 1 and Appendix Table A2). The GMPI was developed using previous quality indices and service‐specific clinical guidelines 1, 2. It includes 28 basic clinical activities across antenatal care (ten items), family planning care (eight items) and sick child care (ten items) based on items asked in all Service Provision Assessment surveys matched with existing clinical guidelines. Six activities are repeated in two or more domains, resulting in 22 discrete clinical activities. Similar to an objective structured clinical examination (OSCE), the resulting index includes essential activities all providers should perform in every clinical visit across countries (and are therefore unweighted), and can serve as a flexible tool to objectively evaluate clinical competency among providers in low‐ and middle‐income settings 19. The index includes items covering history‐taking, physical examination, and counselling actions that should be conducted for all patients regardless of the reason for presentation or the local epidemiology. As history‐taking items included in the index may not apply to antenatal care follow‐up visits, these items were excluded from GMPI calculations for relevant observations; all other index items apply to both first and follow‐up visits across the three service areas. In instances where certain services were provided by a clinician other than the primary provider separate from the actual consultation (e.g. a nurse taking blood pressure measurements prior to the full examination), these activities were recorded as having been performed during the visit. The primary outcome is a Good Medical Practice Index score calculated as an average of the proportion of index items a clinician completed across patient encounters in antenatal care, family planning, or sick child care. The resulting score ranges from 0 to 1 with a higher score corresponding to greater performance of essential clinical actions.

### Covariates

Several factors other than education may influence clinical performance among recent graduates. Using Rowe's framework for explaining health‐worker practices, we identified facility and provider characteristics that corresponded to covariates in the data, and analysed the effects of these on the GMPI 20. Facility characteristics were defined as infrastructure and environmental factors that might be associated with care quality, such as facility management (public or private) and structural quality. Structural quality was measured with an index of service readiness defined by the World Health Organization: general service readiness (50 indicators across five domains: basic amenities, basic equipment, infection prevention measures, diagnostic capacity, and essential medications). Provider characteristics included provider sex, years of pre‐service education, in‐service training, and supportive supervision. In‐service training was defined as receipt of any general in‐service training or in‐service training specific to one of the three service areas within the past six months. Supportive supervision was defined as a health care worker reporting supervision in the last six months that included discussion of problems encountered and receipt of supervisor feedback. We included a covariate for provider type in the model that included all clinical cadres. Finally, we used an indicator variable for each of the nine included countries as a proxy for national factors, including quality of a country's health professions education that may influence quality among providers in the first three years of practice.

### Statistical analysis

To assess quality across items in the Good Medical Practice Index, we calculated the proportion of GMPI clinical items each clinician completed across his or her patient encounters in each service area. We estimated the mean and standard deviation GMPI score for each country and clinician type as an average of mean clinician scores across patient encounters. Clinicians were excluded in countries with fewer than ten providers per cadre. We also calculated 95% confidence intervals for the mean Good Medical Practice Index score of each provider type in each country. The outcome was rescaled to have a mean of zero and a standard deviation of one.

Multivariable models were constructed to test the association of each country with GMPI, controlling for facility and provider characteristics that may influence provider performance and confound the relationship of interest. The association was tested using ordinary least squares linear regression for all clinicians, and then separately among physicians, nurses and midwives, and associate clinicians; models were clustered by facility. Countries were excluded if they had fewer than ten providers in a given clinician cadre. Tanzania, which had a Good Medical Practice score near the median among each clinician type, was used as a reference group for all models. All statistical analyses were carried out using Stata version 14.2 (Stata‐Corp, College Station, TX, USA).

### Ethical approval

The original survey implementers obtained ethical approvals for data collection; the Harvard University Human Research Protection Program deemed this analysis exempt from human subjects review.

## Results

The SPA assessed 6572 of 6755 health facilities across the nine countries of interest; the remainder were closed, empty, inaccessible, or refused assessment. Among assessed facilities, 11 452 clinicians had at least one direct observation of care in one service area. Of these clinicians, 15% had one observation, 25% had two or three observations, 46% had four or five observations, and 14% had six or more observations. The analytic sample is composed of 2223 clinicians in the first three years of practice.”

Table 1 describes characteristics of the 2223 clinicians providing each type of care across the nine countries. Just over half (56%) of clinicians were female, and 51% were in the nurse/midwife category. Physicians and associate clinicians each composed nearly a quarter of the remaining clinicians. A slight majority (52%) of clinicians provided sick child care alone, while 20% provided antenatal care alone, and 10% provided family planning care alone. The vast majority of clinicians (87%) were in their second or third year of practice since completing their health professions education, and 77% were practicing in the surveyed facility by their second year from graduation. Only 17% of clinicians had received relevant in‐service training in the past six months, and 51% received supportive supervision within the same time frame. The number of surveyed clinicians varied across countries, ranging from Uganda with 86 clinicians in the first three years of practice to Tanzania with 620 clinicians. Clinicians largely practiced in public, non‐hospital facilities, such as clinics or health posts, in rural areas. These facilities had a relatively low average structural quality score of 0.67.

**Table 1:** Characteristics of clinicians in the first three years of practice

Variable	*N*	%
	Clinicians (*N* = 2223)	
Clinician characteristics		
Clinician sex		
Female	1238	56
Clinician type		
Physician (MD/Medical officer)	515	23
Associate clinician (e.g. asst. medical officer)	525	24
Nurse/midwife (e.g. registered nurse, nurse midwife)	1144	51
Other (e.g. counsellor, social worker)	39	2
Type of care observed		
Antenatal care	442	20
Family planning	232	10
Sick child care	1147	52
More than one type of care	402	18
Year of practice since completing health education		
First year	275	12
Second year	917	41
Third year	1031	46
In‐service training and supportive supervision		
Any training in relevant service in the past 6 months	384	17
Supportive supervision in the past 6 months^a^	1120	51
Clinician country		
Haiti	289	13
Kenya	149	7
Malawi	280	13
Namibia	142	6
Nepal	312	14
Rwanda	155	7
Senegal	190	9
Tanzania	620	28
Uganda	86	4
Facility characteristics		
Facility type		
Hospital/large health centre	857	39
Non‐hospital (e.g. clinic, health post, dispensary)	1366	61
Urban/Non‐urban		
Urban	570	41
Private/Public		
Private	662	30
Facility structural quality		
Service readiness index^b^ [Mean (SD)]	0.67 (0.16)	
Outcome: Good Medical Practice Index^c^		
Technical quality		
Good Medical Practice score [Mean (SD)]	0.50 (0.20)	

a Supportive supervision is defined as supervision that included feedback and discussion of problems encountered in the past 6 months.

b Service readiness index is a score from 0 to 1 assessing facility preparedness to deliver healthcare based on 50 items in 5 domains: amenities, basic equipment, infection prevention, diagnostic capacity, and essential medicine (WHO SARA report).

c The Good Medical Practice Index is an index of fundamental clinical action items across history‐taking, examination, and counselling that should be performed at every patient visit regardless of service type. See Figure 1 for components of the index.

**Figure 2:**
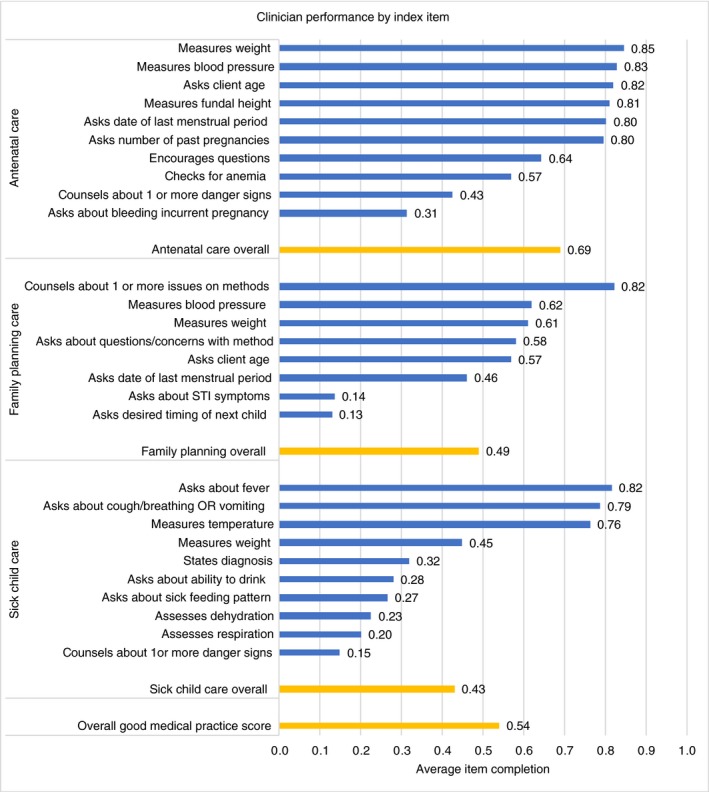
Clinician performance by Good Medical Practice Index item. Raw average of the items is not equal to the average GMP score because the latter is implicitly weighted by frequency of observations of each service type and due to missing values on items. [Colour figure can be viewed at http://wileyonlinelibrary.com]

The overall GMPI score for the sample was 0.50 (SD = 0.20). Item performance varied substantially (Figure 2). Within antenatal care, six of the 10 items had average completions scores at or above 0.80. The provider ‘asks about bleeding in current pregnancy’ had the lowest score at 0.31. Sick child care items scored slightly lower and had a wider range, with only three items scoring over 0.75, and six items falling below 35% completion. Clinicians only counselled parents about one or more danger signs requiring return to the facility 15% of the time. Most family planning index items fell between 45% and 65% completion. However, 82% of women were counselled about one or more issues with one or more family planning methods, and two index items—asking about STI symptoms and asking desired timing of a woman's next child—scored the lowest of all items at 0.14 and 0.13 respectively.

Based on available sample size, Good Medical Practice scores were calculated in all nine countries for nurses and midwives, in five countries for physicians, and five countries for associate clinicians (Table 2). Nurses and midwives had the highest Good Medical Practice score at 0.57 (SD = 0.20), followed by associate clinicians at 0.43 (SD = 0.18), and physicians at 0.42 (SD = 0.16). Good Medical Practice scores also varied by country. On average, Ugandan clinicians performed 0.63 (SD = 0.18) of recommended clinical actions in each service area, followed closely by Kenya at 0.62 (SD = 0.20). Nepal's clinicians had the lowest score at 0.39 (SD = 0.17), indicating that Nepal's 312 clinicians in the first three years of practice performed only 39% of recommended clinical action items on average across service areas.

**Table 2:** Good Medical Practice by country and clinician type (*N* = 2172)^a^^,^ b

	Haiti (2013)	Kenya (2010)	Malawi (2013)	Namibia (2009)	Nepal (2015)	Rwanda (2007)	Senegal (2013–15)	Tanzania (2015)	Uganda (2007)	Overall
Physician^c^										
Mean (SD)	0.43 (0.13)	–	–	–	0.39 (0.17)	0.55 (0.18)	0.37 (0.13)	0.49 (0.19)	–	0.42 (0.16)
*N*	205	–	–	–	172	18	54	54	–	503
Nurse/Midwife^d^										
Mean (SD)	0.49 (0.15)	0.66 (0.18)	0.54 (0.17)	0.60 (0.17)	0.39 (0.15)	0.55 (0.21)	0.48 (0.15)	0.63 (0.23)	0.63 (0.19)	0.57 (0.20)
*N*	81	91	127	141	89	133	111	325	46	1144
Associate Clinician^e^										
Mean (SD)	–	0.55 (0.21)	0.36 (0.15)	–	0.38 (0.20)	–	–	0.42 (0.15)	0.63 (0.17)	0.43 (0.18)
*N*	–	47	151	–	51	–	–	239	37	525
Overall										
Mean (SD)	0.44 (0.14)	0.62 (0.20)	0.44 (0.18)	0.60 (0.17)	0.39 (0.17)	0.55 (0.21)	0.45 (0.16)	0.54 (0.22)	0.63 (0.18)	0.50 (0.20)
*N*	286	138	278	141	312	151	165	618	83	2172

a The Good Medical Practice Index is an index of fundamental clinical action items across history‐taking, examination, and counselling that should be performed at every patient visit regardless of service type. See Figure 1 for components of the index.

b Table excludes clinician type for countries with <10 clinicians sampled in the first three years of practice.

c The physician category includes clinicians such as medical doctors (MDs) and medical officers (MOs).

d The nurse/midwife category includes clinicians such as registered nurses, enrolled nurses, nurse midwives, and auxiliary nurses.

e The associate clinician category includes clinicians such as clinical officers, medical assistants, and clinical technicians.

**Figure 3:**
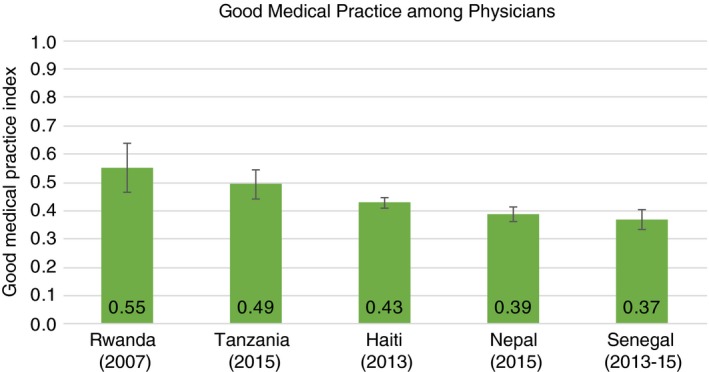
Good Medical Practice among physicians (*N* = 503). The Good Medical Practice Index is an index of fundamental clinical action items across history‐taking, examination, and counselling that should be performed at every patient visit regardless of service type. See Figure 1 for components of the index. Whiskers indicate the 95% confidence interval for mean Good Medical Practice Index score. The physician category includes clinicians such as medical doctors (MDs) and medical officers (MOs). [Colour figure can be viewed at http://wileyonlinelibrary.com]

**Figure 4:**
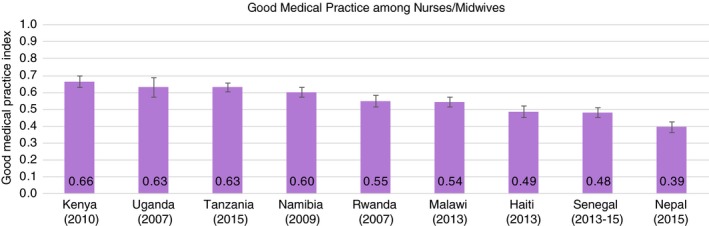
Good Medical Practice among nurses/midwives (*N* = 1144). The Good Medical Practice Index is an index of fundamental clinical action items across history‐taking, examination, and counselling that should be performed at every patient visit regardless of service type. See Figure 1 for components of the index. Whiskers indicate the 95% confidence interval for mean Good Medical Practice Index score. The nurse/midwife category includes clinicians such as registered nurses, enrolled nurses, nurse midwives, and auxiliary nurses. [Colour figure can be viewed at http://wileyonlinelibrary.com]

**Figure 5:**
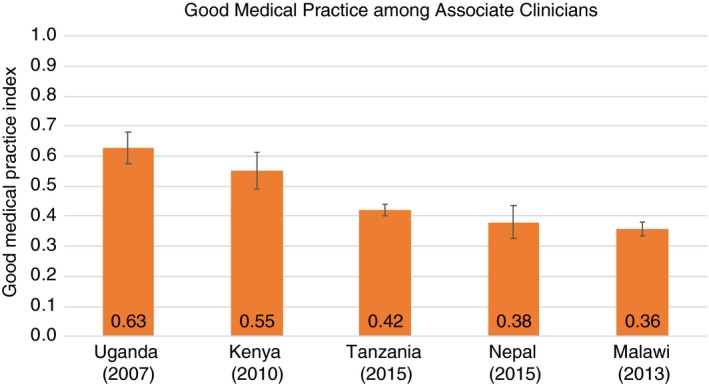
Good Medical Practice among associate clinicians (*N* = 525). The Good Medical Practice Index is an index of fundamental clinical action items across history‐taking, examination, and counselling that should be performed at every patient visit regardless of service type. See Figure 1 for components of the index. Whiskers indicate the 95% confidence interval for mean Good Medical Practice Index score. The associate clinician category includes clinicians such as clinical officers, medical assistants, and clinical technicians. [Colour figure can be viewed at http://wileyonlinelibrary.com]

Among the 503 physicians surveyed across countries, Rwandan physicians had the highest Good Medical Practice score at 0.55 (SD = 0.18), followed by Tanzania, Haiti, Nepal, and Senegal (0.37, SD = 0.13) (Figure 3). Nurses and midwives (*N* = 1144) outperformed physicians in nearly every country (Figure 4); Kenya's nurses and midwives had the highest average score of any clinical cadre in any country at 0.66 (SD = 0.18). Among the 525 associate clinicians, Ugandan clinicians had the highest score at 0.63 (SD = 0.17), followed by Kenya, Tanzania, Nepal, and Malawi (Figure 5). Malawi's associate clinicians were the lowest scoring cadre in any country, completing only 36% of recommended clinical action items on average across patient visits.

**Figure 6:**
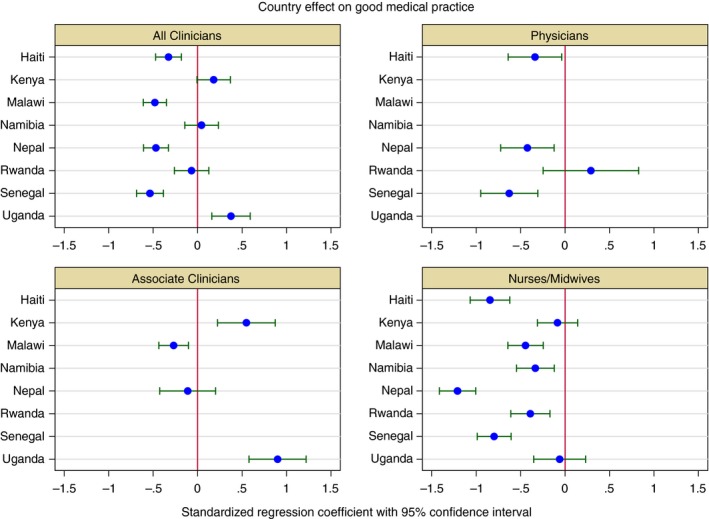
Effect of country on adjusted Good Medical Practice Index score (Reference: Tanzania 2015). The Good Medical Practice Index is an index of fundamental clinical action items across history‐taking, examination and counselling that should be performed at every patient visit regardless of service type. See Figure 1 for components of the index. Good Medical Practice score is rescaled to have a mean of zero and a standard deviation of one. Estimates were obtained using ordinary least squares regression clustered at the facility level. All models were adjusted for facility structural quality, management type, provider sex, years of education, training and supportive supervision. The all‐clinician model was also adjusted for provider type. Twenty‐two providers were excluded from the models due to missingness for at least one covariate. [Colour figure can be viewed at http://wileyonlinelibrary.com]

Figure 6 and Appendix Table A3 present the results of the fully adjusted multivariable regression models, focusing on the effect of country on GMPI. The analytical sample included 2150 clinicians composed of 497 physicians, 1132 nurses and midwives, and 521 associate clinicians with complete data on covariates. We found that clinicians from Uganda and Kenya have higher Good Medical Practice scores on average than Tanzania. The best performer, Uganda, was 0.38 standard deviations higher than Tanzania (95% CI 0.16, 0.59), which equates to the completion of approximately one additional clinical action item on average. Nepal, Malawi, and Senegal, the lowest performers, completed approximately one clinical action item less than Tanzanian clinicians on average. Among physicians, only Rwandan clinicians performed more clinical action items than Tanzania, but this was not a statistically significant difference; Haiti, Nepal, and Senegal performed significantly lower than Tanzania, with Senegal 0.63 standard deviations below the reference (95% CI −0.95, −0.31). Among nurses and midwives, all countries performed more poorly than Tanzania (though coefficients for Uganda and Kenya were not statistically significant); nurses and midwives in Nepal performed over one full standard deviation lower (β = −1.21, 95% CI −1.41, −1.01). Finally, associate clinicians in Uganda and Kenya had Good Medical Practice scores that were significantly higher than Tanzania, and Malawi significantly lower. Uganda, whose associate clinicians were top performers, scored 0.90 standard deviations higher than Tanzania (95% CI 0.58, 1.22).

## Discussion

We assessed clinical performance among recent clinical graduates providing primary care in nine low‐ and middle‐income countries and found that on average clinicians are completing fewer than half of the clinical actions for antenatal care, family planning, and sick child care. This is concerning as the Good Medical Practice Index represents the most basic items required for assessment or differential diagnosis and assignment of appropriate treatment. For example, performance on these core items is only slightly better than on more demanding metrics of performance (i.e. adherence to guidelines) in antenatal care, and even lower in sick child care 1, 2, 3. Overall, quality of care is worse for sick child care compared to antenatal care or family planning services 1, 21. This study benefits from the use of large, nationally representative samples or censuses that included direct observations of care for multiple provider and service types, allowing for comparison of quality performance both within and across countries.

Higher clinical qualifications did not guarantee superior quality performance. Nurses and midwives outperformed physicians and associate clinicians in most countries, completing approximately one to two additional clinical actions on average across patient visits than other clinician types 22, 23. Nursing and midwifery practice, though, remains suboptimal, with a Good Medical Practice score of only 0.57. Many countries are investing in the associate clinician cadre, such as clinical officers and assistant medical officers, to expand access. Previous evidence on quality of care provided by associate clinicians is mixed 24, 25. In this study, associate clinicians provided the poorest quality among the three clinical cadres in nearly every country. Differences in service quality between cadres may be due to differences in emphasis during training or infrequent provision of certain services, such as family planning care by physicians; however, the overall poor performance of providers in the study suggests that training deficiencies exist for all cadres.

There were statistically significant differences in performance across countries. These persisted after controlling for factors that influence health system quality and investment, such as facility structural quality, in‐service trainings, and supportive supervision. While some countries with higher average quality, such as Kenya and Uganda, outperformed countries with lower average quality across all three clinical cadres, certain national cadres diverged from a nation's overall performance. For example, Tanzania ranks highly in physician and nurse/midwife performance, but performs at the median for associate clinicians. While overall scores were low, we identified particular deficits in patient counselling, suggesting a need for increased patient‐centred education. Differences between countries and providers may be due to several factors, such a selection of candidates, which may be influenced by quality of secondary education, quality of clinical education for each cadre, and length and quality of clinical exposure during training.

Studies of clinical training programmes have identified several challenges, including a lack of practical exposure, poor standardisation of curricula, insufficient quality assurance systems, pathology‐based training models, and failure to emphasise acquisition of clinical skills 26, 27, 28. Some nations lack standardised competency verification procedures beyond graduation to ensure fitness to practice, or procedures for ongoing competency assessment 13. As governments expand health professions education to increase human resources for health, the quality of education offered by strained systems may weaken further 29. In particular, lack of infrastructure and resources for teaching and learning limits institutional capacity to provide consistently high quality instruction to students and to innovate in clinical education 30. Reforming health professions education to address these deficits is a challenge in many nations due to scarce resources, yet it must become a greater priority for improving and sustaining the health care workforce in low‐ and middle‐income countries 10, 31.

The Good Medical Practice Index, while a conservative measure of clinical performance, underscores the importance of evaluating fundamental clinical performance across various types of care and ensuring clinicians are well‐prepared for practice. In 2010, The Lancet Commission on Health Professionals for a New Century called for increased use of competency‐based education to ensure high quality medical practice for all providers 11. Given our limited knowledge of clinical competence in LMICs, their recommendations have been largely unfulfilled; however, they remain highly relevant given the findings of this and other studies. One approach to improve clinical performance is to use standardised patients, actors trained to present with specific medical conditions, which can be used as part of graduating or licensing requirements. Many countries have adopted objective structured clinical examinations (OSCEs) using standardised patients to assess competence before graduation 19. OSCEs test a broad range of clinical skills including problem‐solving, communication, decision‐making, and patient management abilities. By contrast, written examinations test only cognitive knowledge, which is only one aspect of competence. These simulations of clinical practice have been found to be reliable and valid, although costly 32, 33, 34, 35. Adaptation of OSCEs and other tools to evaluate clinical performance for low‐income settings should be a global priority.

Data used in this study were based on large, nationally representative samples of facilities or facility censuses from each country; clinical actions were recorded by trained observers, a gold standard in quality measurement. However, this study is subject to some limitations. Sample size was small in certain clinical cadres, limiting precision of estimates. Data were collected by trained observers, which could lead clinicians to behave differently (Hawthorne effect), and is subject to observer error. Other studies that removed the first observation from analysis found similar performance 36. Hawthorne effect would bias our results upward, suggesting that actual practice may potentially be worse than observed here, thus strengthening the concern about pre‐service education. There may also be residual confounding in regression estimates from unobserved variables, especially at the facility and/or national levels, which may impact a clinician's quality performance such as leadership and governance. Furthermore, as many facilities in the sample have only one clinician in the first three years of practice, our ability to test the impact of facility characteristics on clinical performance across providers is limited. This analysis uses broad clinical cadres for analysis; more granular categories, such as separation of nurses and midwives, would aid analysis. Given differences in clinician categorisation across Service Provision Assessments and varying clinical responsibilities across countries, we were unable to further disaggregate these categories. We were also unable to provide a comparison with recently graduated providers in high‐income countries which limits inference. Finally, the GMPI assesses clinical performance by processes of care, which is only one component of clinical competency; a full assessment of competency would also include how well an activity was executed to determine whether differential performance between clinical cadres impacts health outcomes. However, the objective of this study was to identify a readily measurable set of clinical items that could be used to assess clinical performance across a range of services and providers. Given our limited knowledge of clinical performance in low‐ and middle‐income countries, the GMPI remains an important contribution to understanding global clinical quality.

This study demonstrates overall poor clinical performance in outpatient care for primary care conditions among clinicians in the first three years of practice. While many low‐ and middle‐income countries focus on the number and distribution of available providers, our results highlight a significant opportunity to improve quality and health outcomes through a focus on health professions education. Renewed attention and innovative approaches, including the use of objective evaluative tools and increased competency‐based education, may provide an opportunity to better prepare clinicians for practice and ensure a high standard of care from every clinician in every patient encounter.

## Declaration

The sponsor of this study had no role in the study design, data collection, data analysis, data interpretation, writing of the report, or the decision to submit this report for publication. The corresponding author had full access to all the data used in the study and final responsibility for the decision to submit for publication.

## Appendix 1

**Table A1:** Service Provision Assessment provider types by country

Category	Haiti	Kenya	Malawi	Namibia	Nepal
Physicians	Generalist doctors	Specialist	Generalist (non‐specialist)	Specialist (including pathologist)	Generalist (non‐specialist)
	Generalist surgeons	Medical officer	Specialist medical doctors	Medical officer (physician)	Obgyn
	Specialist doctors				Anaesthesiologist
					Pathologist
					General surgeon
					Pediatrician
					Other specialists (medical doctors)
					Medical officer
Associate clinicians		Clinical officer	Clinical officer		Health assistant/public health inspector
			Medical assistant		
			Clinical technician		
Nurses/midwives	Nurse	BSN nurse	Registered nurse	Registered nurse/midwife	Anaesthetic assistant
	Nurse/midwife	Registered nurse	Registered nurse midwife	Enrolled nurse/midwife	Nurse or auxiliary nurse midwife
	Auxiliary nurses	Registered midwife	Registered psychiatric nurse	Nurse assistant/auxiliary	
		Enrolled nurse	Registered nurse with diploma		
		Enrolled midwife	Enrolled nurse		
		Nurse aide	Enrolled midwife/nurse midwife technician		
			Enrolled nurse midwife		
			Community health nurse		
Other	Pharmacist	Laboratory technologist	Laboratory technologist	Pharmacist	Pharmacist
	Pharmacy assistant	Laboratory technician/assistant	Laboratory technician	Pharmacist assistant	Laboratory technologist/officer/technician/assistant
	Laboratory technician	Nutritionist/nutrition technician	Laboratory assistant	Lab scientist	Radiographer/dark room assistant
	Dental laboratory technician	Health education officer	Radiographer	Lab technologist	Physiotherapist/physiotherapy assistant
	Dental hygienist	Social worker	Environmental health officer	Lab technician	Counsellor with clinical qualification
	Auxiliary dentist	HIV counsellor/lay counsellor	Health surveillance assistants	Medical assistant	Counsellor without clinical qualification
	Other community health workers	Public health officer	HTC counsellors	CHW/home‐based caregiver	Other clinical staff not listed above
	Radiology technician	Public health technician	No technical qualification	Occupational therapist	Non‐clinical staff/no technical qualification
	Medical imagery technician	No technical qualification	Other	Physiotherapist	
	Non‐technical qualification	Other		Social worker	
	Other			Medical rehab officer/worker	
				Nutritionist	
				Community HIV counsellor	
				Lifestyle ambassador (TB/HIV)	
				Field promoter (TB/HIV)	
				Health inspector	
				Environmental health assistant	
				Other	

Category	Rwanda	Senegal	Tanzania	Uganda
Physicians	Gyneco‐OB	Generalist doctors	Generalist (non‐specialist) medical doctors	Consultant
	Pediatrician	Generalist surgeons	Specialists medical doctors)	Medical officer
	Surgeon	Specialist doctors (2014 only)	Anaesthetist	
	Other medecin specialist			
	Medecin generalist			
	Medical officer			
	Radiologist			
	Anaesthetist a1			
Associate clinicians			Assistant medical officer	Clinical officer
			Clinical officer	
			Assistant clinical officer	
Nurses/midwives	Nurse a1	Nurse (includes state nurse, bloc nurses and anaesthetists)	Registered nurse (including nursing officers and midwives)	Registered nurse
	Midwife a1	Midwife	Enrolled nurse (including trained nurses and public health nurse)	Registered midwife
	Nurse a2	Assistant infirmier	Nurse assistant/attendant	Public health nurse
	Nurse a3	Matrone (2014 only)		Enrolled nurse
				Enrolled midwife
				Comprehensive nurse
				Nursing assistant
				Nursing aide
Other	Pharmacist a0	Laboratory technician	Pharmacist	Pharmacist
	Pharmacist a1	Dental technician	Pharmaceutical technician	Pharmacy dispenser
	Pharmacy lab tech a1	Technicien superieur de radiologie	Pharmaceutical assistant	Laboratory technologist
	Pharmacy lab tech a2	Technicien superieur en anaesthesie/reanimation	Laboratory scientist	Laboratory technician
	Pharmacy lab tech a3	Technicien superieur en imagerie medicale	Laboratory technologist	Laboratory assistant
	Dentist a1	Technicien superieur en othopedie	Laboratory technician	Social worker
		Technicien superieur en ophtamologie		
	Auxiliary health worker	Technicien superieur en genie sanitaire	Laboratory assistant	HIV/AIDS counsellor
	Asst. social a0	Biologist	Other	Other counsellor
	Asst. social a1	Relais		Health educator
	Asst. social a2	Autres agent de santé communautaire		Nutritionist
	Nutritionist a1	Technicien superieur en administration		Pathologist
	Nutritionist a2	Technicien superieur de maintenance		Other staff providing client services
	Hygiene & assainissement a1	Assistant lab technician (2013 only)		Statistician
	Physiotherapist	Qualification non‐technique (2013 only)		Records clerk
	Management	Other		Hospital administrator
	Technical support staff			Other non‐client
	Management support staff			
	Other			

**Table A2:** Components of the Good Medical Practice Index

Type of service	Clinical action of health care provider
Antenatal care	
History‐taking	Asks client age (first visit only) Asks number of past pregnancies (first visit only) Asks date of last menstrual period (first visit only) Asks if client has bleeding in current pregnancy
Examination	Checks for anemia Measures fundal height Measures blood pressure Measures weight
Counselling	Encourages questions Counsels about ≥1 danger signs for return consultation
Family planning	
History‐taking	Asks client age Asks desired timing of next child Asks about STI symptoms Asks date of last menstrual period
Examination	Measures blood pressure Measures weight
Counselling	Asks about questions/concerns with current method Counsels about ≥1 issues on ≥1 methods
Sick child care	
History‐taking	Asks about ability to drink Asks about fever Asks about sick feeding pattern Asks about cough/difficulty breathing OR vomiting
Examination	Measures temperature Assesses dehydration Assesses respiration Measures weight
Counselling	States diagnosis Counsels about ≥1 danger signs for return consultation

**Table A3:** Results of multivariable regression models of good medical practice by clinician type (Reference: Tanzania 2015)^a^^**,**^ b ^**,**^ c

	All Clinicians	Physicians	Nurses/Midwives	Associate Clinicians
Haiti (2013)	−0.33*** (−0.47, −0.18)	−0.34** (−0.64, −0.04)	−0.84*** (−1.07, −0.62)	–
Kenya (2010)	0.18* (−0.01, 0.37)	–	−0.09 (−0.31, 0.14)	0.55*** (0.22, 0.87)
Malawi (2013)	−0.48*** (−0.61, −0.35)	–	−0.45*** (−0.64, −0.25)	−0.27*** (−0.44, −0.10)
Namibia (2009)	0.04 (−0.14, 0.23)	–	−0.33*** (−0.55, −0.12)	–
Nepal (2015)	−0.47*** (−0.61, −0.33)	−0.42*** (−0.72, −0.12)	−1.21*** (−1.41, −1.01)	−0.11 (−0.43, 0.20)
Rwanda (2007)	−0.07 (−0.26, 0.13)	0.29 (−0.25, 0.83)	−0.39*** (−0.61, −0.17)	–
Senegal (2013–15)	−0.54*** (−0.69, −0.39)	−0.63*** (−0.95, −0.31)	−0.80*** (−0.99, −0.61)	–
Uganda (2007)	0.38*** (0.16, 0.59)	–	−0.06 (−0.08, 0.21)	0.90*** (0.58, 1.22)
Observations	2150	497	1132	521
*R*‐squared	0.22	0.11	0.19	0.21

95% CI in parentheses.

a The Good Medical Practice Index is an index of fundamental clinical action items across history‐taking, examination, and counselling that should be performed at every patient visit regardless of service type. See Figure 1 for components of the index.

b Good Medical Practice score is rescaled to have a mean of zero and a standard deviation of one.

c Estimates were obtained using ordinary least squares regression clustered at the facility level. All models were adjusted for facility structural quality, management type, provider sex, years of education, training, and supportive supervision. The all‐clinician model was also adjusted for provider type. Twenty‐two providers were excluded from the models due to missingness for at least one covariate.

****P* < 0.01, ***P* < 0.05, **P* < 0.1
